# The impact of urban spatial environment on COVID-19: a case study in Beijing

**DOI:** 10.3389/fpubh.2023.1287999

**Published:** 2024-01-08

**Authors:** Zhen Yang, Jiaxuan Li, Yu Li, Xiaowen Huang, Anran Zhang, Yue Lu, Xu Zhao, Xueyan Yang

**Affiliations:** School of Architecture and Urban Planning, Beijing University of Civil Engineering and Architecture, Beijing, China

**Keywords:** epidemic, urban spatial environment, COVID-19, geographically weighted regression, binomial logistic regression

## Abstract

Epidemics are dangerous and difficult to prevent and control, especially in urban areas. Clarifying the correlation between the COVID-19 Outbreak Frequency and the urban spatial environment may help improve cities’ ability to respond to such public health emergencies. In this study, we firstly analyzed the spatial distribution characteristics of COVID-19 Outbreak Frequency by correlating the geographic locations of COVID-19 epidemic-affected neighborhoods in the city of Beijing with the time point of onset. Secondly, we created a geographically weighted regression model combining the COVID-19 Outbreak Frequency with the external spatial environmental elements of the city. Thirdly, different grades of epidemic-affected neighborhoods in the study area were classified according to the clustering analysis results. Finally, the correlation between the COVID-19 Outbreak Frequency and the internal spatial environmental elements of different grades of neighborhoods was investigated using a binomial logistic regression model. The study yielded the following results. (i) Epidemic outbreak frequency was evidently correlated with the urban external spatial environment, among building density, volume ratio, density of commercial facilities, density of service facilities, and density of transportation facilities were positively correlated with COVID-19 Outbreak Frequency, while water and greenery coverage was negatively correlated with it. (ii) The correlation between COVID-19 Outbreak Frequency and the internal spatial environmental elements of neighborhoods of different grades differed. House price and the number of households were positively correlated with the COVID-19 Outbreak Frequency in low-end neighborhoods, while the number of households was positively correlated with the COVID-19 Outbreak Frequency in mid-end neighborhoods. In order to achieve spatial justice, society should strive to address the inequality phenomena of income gaps and residential differentiation, and promote fair distribution of spatial environments.

## 1 Introduction

Currently, humanity has entered a risk society, where major public health events can have a significant impact on a country’s social, economic, and political order, jeopardizing national security and development. The COVID-19 pandemic has had a profound impact on lifestyle and the way urban spaces are used. Researching the spatial distribution of COVID-19 in cities and its driving mechanisms can help uncover the transmission mechanisms and diffusion patterns of the epidemic, providing a theoretical basis for policy design (1).

The various factors related to epidemic outbreaks are complex, and many scholars have conducted research on the spatial distribution and transmission patterns of COVID-19 from social, economic, climate, and population perspectives (2–4). Some studies have found that socioenvironmental factors, including seven variables such as the internet development index and literacy index, are related to the spatial differentiation of COVID-19 (5). There are also studies that focus on the association between meteorological factors such as air temperature, wind speed, precipitation, and the spatial distribution of COVID-19 cases (6). Some scholars believed that many demographic factors are significantly correlated with the spread of COVID-19, such as population density and human mobility (7, 8). Many studies have demonstrated that the disease was more concentrated in central areas with high population density and dense urban land use (9).

In addition, different urban forms and design factors can affect the dynamics of epidemics. At the urban level, some studies have shown that urban spatial environmental factors such as diversity, destination accessibility, distance to transit, design, and density are spatially consistent with the spread of COVID-19 (10). Some scholars have also compared the differences in the impact of density and connectivity on the spatial proliferation of COVID-19 (11). These factors may affect the spread of the disease by influencing people’s patterns of interaction and spatial usage. At the community level, some scholars believe that socio-economic factors and community building environments have varying degrees of impact on the outbreak, spread, and residents’ health condition and health behaviors related to COVID-19 (12–14). For example, factors such as community economic status, housing conditions, and medical resources may affect people’s sensitivity to and ability to respond to the disease. However, there are also studies showing that there is no clear conclusion about any association between compact neighborhood design and the transmission of infection, and further research is needed (15).

Currently, research on urban factors that influence the outbreak of COVID-19 mainly focuses on the level of building environment, and does not fully consider other urban spatial environmental factors that affect the transmission of COVID-19, such as urban green spaces, water bodies, and service facilities. There has also been limited exploration of the correlation between built environment elements within communities and the outbreak of COVID-19, which still has certain limitations.

On the other hand, current research on the environmental impact of COVID-19 often obtains data through questionnaire surveys, and uses quantitative methods such as multiple linear regression, logistic regression, stepwise regression, and factor analysis to determine the factors that influence the outbreak and spread of COVID-19 (16, 17). These traditional quantitative methods may neglect the spatial differences in factors and regression relationships. Therefore, some researchers have conducted spatial heterogeneity analysis using geographically weighted regression or studied spatial spillover effects using spatial econometric models (18–20). This requires researchers to obtain continuous and measurable data within a certain spatial range.

In addition, some scholars have focused on the impact of various factors on the COVID-19 mortality rate. Using methods such as ordinary least squares, spatial econometric models, geographically weighted regression, and machine learning, they have analyzed population factors such as population density, age, and ethnicity, socio-economic factors such as household income, education, and rent, as well as individual health factors such as chronic diseases, obesity, and unhealthy lifestyle habits, on the relationship between COVID-19 mortality rate (21–23).

Therefore, this study aims to explore the correlation between the frequency of COVID-19 outbreaks and urban spatial environmental factors such as density, environment, and facilities, as well as the relationship with internal spatial environmental factors within communities such as housing prices, building age, number of buildings, and number of households. Our research results can contribute to a better understanding of the spatial environmental factors that influence the transmission of epidemics and have significant implications for improving urban resilience in responding to public health emergencies.

## 2 Materials and methods

### 2.1 Selection of indicators

Research on the factors influencing the spread of the pandemic has been extensive, and there is a general consensus on the conclusions. Factors such as population mobility, population density, income, ecological environment quality, and urban built environment differences have been found to be correlated with the spread of the disease. Furthermore, studies have shown that urban geometry plays a more significant role in influencing COVID-19 incidence rates than sociodemographic characteristics (24). Refer to the selection of urban spatial environmental indicators in other studies and incorporate internal environmental indicators (25–27). This study aims to comprehensively analyze the correlation between various influencing indicators and the COVID-19 pandemic, and provide valuable supplements to existing research.

The urban spatial environment was divided into two dimensions: External environmental elements and Internal environmental elements. The external environment was characterized in terms of six elements: Building Density (BD), Volume Ratio (VR), Water and Greenery Coverage (WGC), Density of Commercial Facilities (DCF), Density of Public Service Facilities (DSF), and Density of Transportation Facilities (DTF). Building density and floor area ratio can represent the development intensity of a city, while water bodies and green coverage can reflect the ecological environment. The density of commercial facilities, transportation facilities, and public facilities can indicate the correlation between the concentration of the population in public service facilities and the outbreak of the pandemic.

At the same time, the internal environment was characterized in terms of four elements: Housing Price (HP), Building Age (BA), Number of Buildings (NB), and Number of Households (NH). Housing prices and building age can partly indicate the economic conditions of the community, while the number of buildings and the number of households can represent the concentration of people in the community (Table 1).

**Table 1:** Selection of indicators and data source.

First-level indicator	Second-level indicator	Third-level indicator	Unit of measurement	Source
Urban spatial environment	External spatial environmental	Building Density (BD)	%	https://www.amap.com/
		Volume Ratio (VR)	%	https://www.amap.com/
		Water and Greenery Coverage (WGC)	%	https://www.gscloud.cn/
		Density of Commercial Facilities (DCF)	per 1/4 square kilometer	https://www.amap.com/
		Density of Service Facilities (DSF)	per 1/4 square kilometer	https://www.amap.com/
		Density of Transportation Facilities (DTF)	per 1/4 square kilometer	https://www.amap.com/
	Internal spatial environment	Housing Price (HP)	RMB¥	https://www.amap.com/
		Building Age (BA)	Year	https://www.amap.com/
		Number of Buildings (NB)	Units	https://www.amap.com/
		Number of Households (NH)	Units	https://www.amap.com/
COVID-19 outbreak frequency			Times	https://data.beijing.gov.cn/index.htm http://wjw.beijing.gov.cn/wjwh/ztzl/xxgzbd/gzbdyqtb/index.html https://www.bjd.com.cn/index.shtml

### 2.2 Data sources

According to the Prevention and Control Program for COVID-19 Pneumonia (9th Edition) issued by China’s National Health and Health Commission, to better prevent and control the source of infection, home health surveillance was required to be conducted for 7 days after COVID-19 patients had recovered and been discharged from hospital. In addition, the absence of new infections for 7 consecutive days in medium- and high-risk areas was considered one of the requirements for the release of risk areas. In Beijing, the outbreak records and announcements are basically based on a 7-day cycle, and new patients in the affected areas will not be announced within 7 days. The lockdown will also be lifted after 7 consecutive days with no new cases reported. Therefore, this study used a 7-day cycle to record the location of new confirmed cases living in within Beijing’s fifth ring road for a total of 53 cycles over approximately 1 year from November 1, 2021 to November 1, 2022, by searching the Beijing Municipal Government Data Resource Network,^1^ the Beijing Municipal Health and Wellness Commission,^2^ and the Beijing Daily.^3^ Using ArcGIS, the above statistical information corresponded to 2,769 rectangular cells within the study area of 500 m × 500 m. The frequency of outbreaks in each rectangular cell was calculated as the mean ratio of the cycles in which new cases were recorded or classified as medium- to high-risk areas to all 53 cycles in each rectangular cell. It has been confirmed that meteorological factors caused by seasonal changes can affect the transmission of COVID-19, but there was currently a large discrepancy in the conclusions of relevant research, and the fundamental reasons for the discrepancy were not yet clear (28). For example, some studies suggest that high temperatures could limit the spread of COVID-19 (29–31), while others believed that a decrease in temperature is negatively correlated with the spread of COVID-19 (32). Therefore, this study used 1 year of epidemic data, which could avoid the impact of seasonal weather factors on research results.

Data on BD, VR, DCF, DPSF, DTF, NB, NH, HP and BA were obtained from Gaode Map.^4^ In this study, ArcGIS is used to calculate the building density and plot ratio based on building outlines and building height data, and the density is calculated based on the quantity of various public service facilities. Data on WGC were obtained from the Landset-8 satellite remote sensing map in the Chinese Academy of Sciences Geospatial Data Cloud for July 2020.^5^ The supervised classification tool of ArcGIS is used to extract the water area and green space coverage range and further calculate their area and coverage ratio.

### 2.3 Evaluation methodology

**Figure 1:**
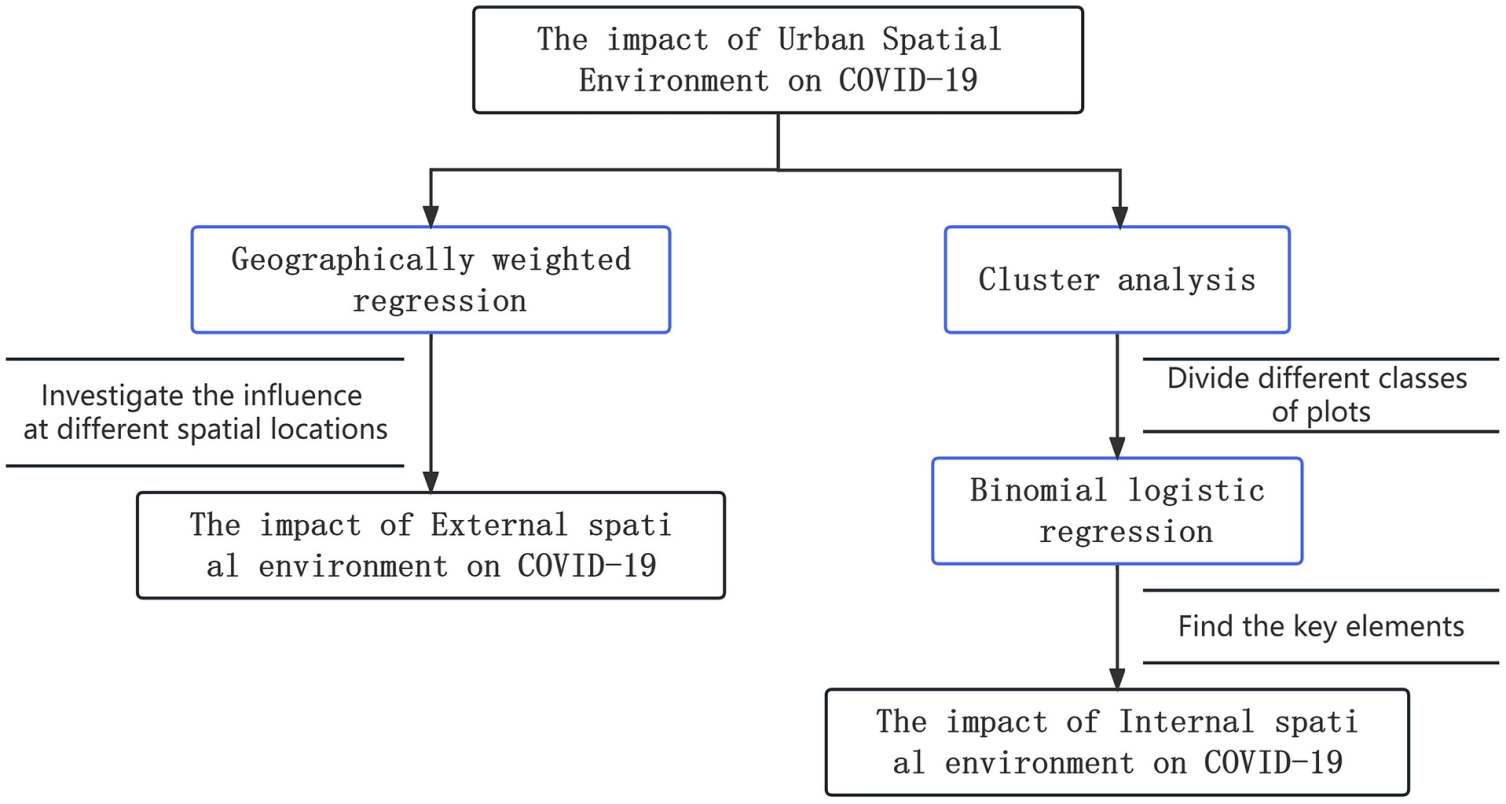
Evaluation methodology.

This study aims to investigate the mechanism of the impact of urban spatial environment on the frequency of COVID-19 outbreaks. Firstly, geographically weighted regression is used to explore the role of external spatial environmental factors in COVID-19 and their spatial variations. Then, cluster analysis is applied to classify the affected communities, followed by logistic regression to examine the influence of internal environmental factors on the frequency of COVID-19 outbreaks (Figure 1).

#### 2.3.1 Geographically weighted regression

In this study, Geographically Weighted Regression (GWR) was used to investigate the influence of urban spatial environmental elements on the COVID-19 outbreaks frequency at different spatial locations.

GWR is an extended model of multiple linear regression, which can create a local regression equation for each point in the range of the model. GWR introduces a spatial weight function to estimate the different relationships between variables in different regions based on spatial variability to better characterize the quantitative relationships’ spatial variation (33). The GWR equation is as follows (Eq. 1):


Eq.1 $$y_{i}=\beta_{0}\left(u_{i},v_{i}\right)+\overset{m}{\underset{k=1}{\sum }}\beta_{k}\left(u_{i},v_{i}\right)X_{ik}+\varepsilon_{i}$$

where $y_{I}$ is the COVID-19 outbreak frequency at spatial position $\left(u_{i}{\mathrm{,v}}_{i}\right)$ , $X_{\mathrm{ik}}$ denotes the BD, VR, WGC, DCF, DPSF, DTF at spatial position $\left(u_{i}{\mathrm{,v}}_{i}\right)$ , $\beta_{0}\left(u_{i}{\mathrm{,v}}_{i}\right)$ is the intercept term of the regression relationship, $\beta_{k}\left(u_{i}{\mathrm{,v}}_{i}\right)$ is the regression coefficient of the kth independent variable at spatial position $\left(u_{i}{\mathrm{,v}}_{i}\right)$ , which is a continuous function of spatial position $\left(u_{i}{\mathrm{,v}}_{i}\right)$ , and $\varepsilon_{i}$ is a mutually independent random error term.

#### 2.3.2 Cluster analysis

In this study, clustering analysis was used to study the division of different clusters of neighborhoods that had been affected by COVID-19 epidemic to subsequently study the relationship between the frequency of epidemic outbreaks in different clusters of neighborhoods and the environmental elements within each cluster of neighborhoods.

Cluster analysis is an important computational method in data mining, which uses the relationships between sample data variables to represent the relationships between samples. Through clustering, the same or similar objects can be classified into a cluster, and the average center of objects belonging to the same cluster can be taken as the center of the cluster. The cluster centers can reflect the common properties of the objects in the cluster. The relationships between cluster centers can be calculated to determine the difference between different clusters (34, 35).

#### 2.3.3 Binomial logistic regression

In this study, Binomial Logistic Regression (BLR) was used to find the key environmental elements that influence the frequency of epidemic outbreaks.

BLR is a classification model represented by a conditional probability distribution P(Y|X) in the form of a parametric logistic distribution. Here, the random variable X takes the value of a real number, and the random variable Y takes the value of 1 or 0. We classified the presence or absence of an epidemic as 1 or 0. The binomial logistic regression model was the following conditional probability distribution (36, 37) (Eqs. 2, 3).


Eq.2 $$P\left(Y=1|x\right)=\frac{\exp\left(w\;\;\cdot \;x+b\right)}{1+\exp\left(w\;\cdot \;x+b\right)}$$

Eq.3 $$P\left(Y=0|x\right)=\frac{1}{1+\exp\left(w\;\cdot \;x+b\right)}$$

Here, x ∈ Rn is the input, Y ∈ (1) is the output, w ∈ Rn and b ∈ R are the parameters, w is called the weight vector, b is called the bias, and w∙x is the inner product of w and x.

For a given input instance x, P(Y = 1|x) and P(Y = 0|x) can be found according to Eqs. 2 and 3. The logistic regression compares the magnitude of the two conditional probability values and assigns the instance x to the class with the larger probability value.

For convenience, the weight vector and the input vector can be expanded; they are still denoted as w, x, i.e., w = (w(1),w(2),...,w(n),b)T, x = (x(1),x(2),...,x(n),1)T. The logistic regression model is then as follows (Eqs. 4, 5):


Eq.4 $$P\left(Y=1|x\right)=\frac{\exp\left(w\;\cdot \;x\right)}{1+\exp\left(w\;\cdot \;x\right)}$$

Eq.5 $$P\left(Y=0|x\right)=\frac{1}{1+\exp\left(w\;\cdot \;x\right)}$$

## 3 Results and analysis

### 3.1 Correlation analysis of COVID-19 outbreak frequency and external spatial environment

#### 3.1.1 Analysis of urban spatial environmental characteristics

In the analysis of urban spatial environmental characteristics, the main urban spatial elements studied comprised BD, VR, WGC, DCF, DSF, and DTF. ArcGIS was used to establish 500 m × 500 m rectangular cells covering the study area, and the building density and volume ratio within the rectangular cells were calculated using building outline and building height data from Gaode Map. In addition, we calculated the density of commercial facilities, service facilities, and transportation facilities. Based on a Landset-8 satellite remote sensing map for July 2020 from the Geospatial Data Cloud of the Chinese Academy of Sciences, surface cover extraction was performed using the supervised classification method to calculate the coverage of waters and greenery within the rectangular cells.

The BD and VR of the study area showed a spatial pattern of high in the middle and low in the surrounding areas. The areas with the highest BD were mainly concentrated within the second ring road, or in the northeastern and northwestern parts of Beihai Park; there were more points with high BD scattered around the southern fifth ring. The areas with higher VR were scattered between the second and fourth rings, while the overall VR in the old city within the second ring and outside the fourth ring were lower.

The spatial pattern of WGC in the study area was low in the central part and high in the surrounding areas, which was opposite to the spatial distribution pattern of BD and VR in the study area. The areas with the highest levels of WGC were the Summer Palace and Olympic Forest Park near the North 5th Ring Road, the Nanyuan Forest Wetland Park and several country parks near the South 5th Ring Road, followed by the Chaoyang Park area near the Northeast 4th Ring Road, the Lize Financial and Business District near the Southwest 3rd Ring Road, and the Temple of Heaven Park and the Six Seas area near the 2nd Ring Road.

**Figure 2:**
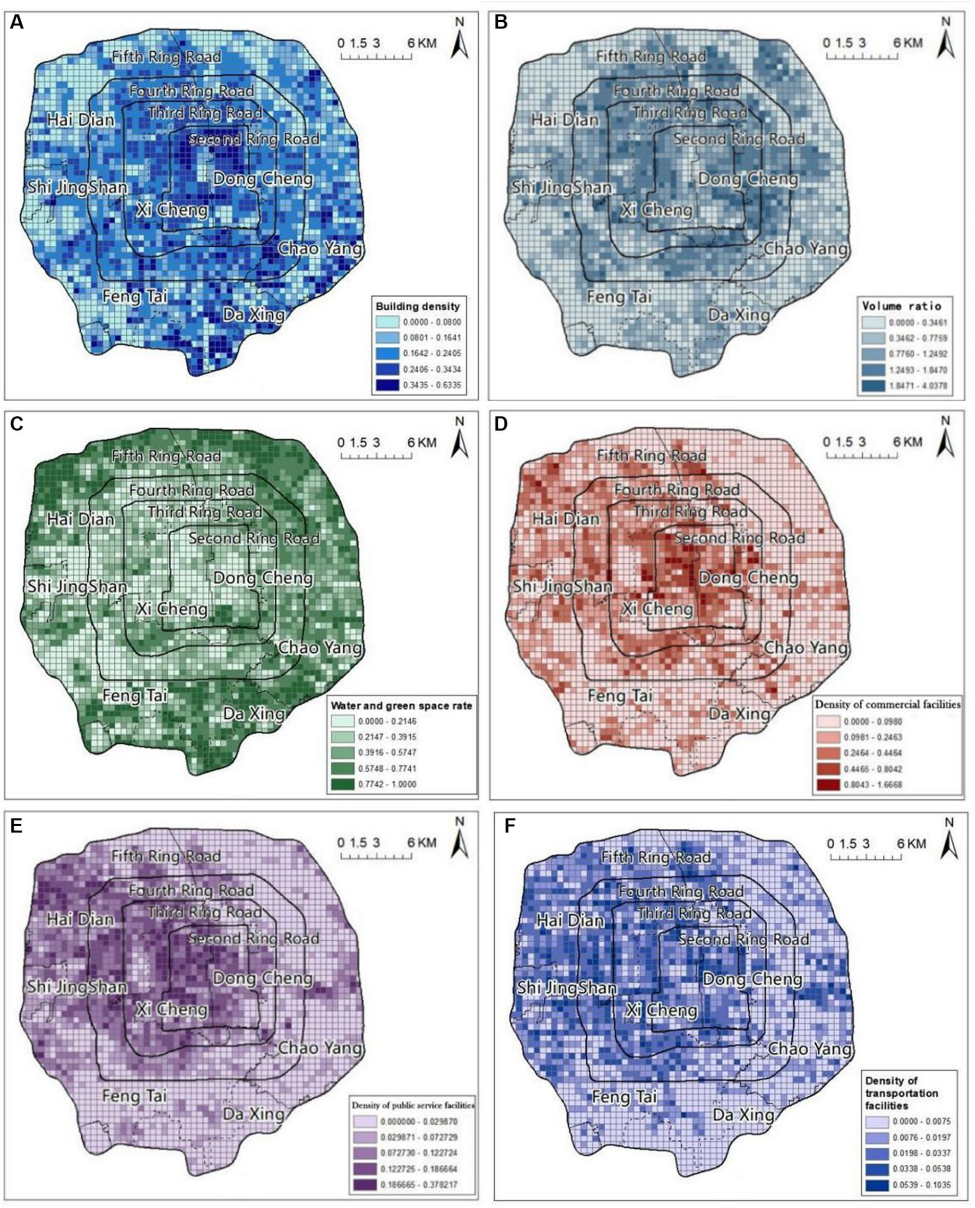
Results of analysis of urban spatial environmental characteristics BD **(A)**, VR **(B)**, WGC **(C)**, DCF **(D)**, DPSF **(E)**, DTF **(F)**.

The DCF and DSF in the study area showed a spatial pattern of high density in the northwest, a scattered distribution in the southeast, and low density in other areas. DCF and DSF facilities were mainly concentrated in Xicheng District, Dongcheng District and Haidian District, and the areas with the highest facility rates were scattered within the second ring road and the northwest section between the third and fourth ring roads. The next highest density of DSF was in the Chaoyang District near the East 4th Ring Road, which showed a distribution pattern extending eastward. Areas with high-DCF and high-DSF were scattered in a dotted pattern near the fifth ring road in the southeast. The DTF was also higher in the northwest, lower in other areas, and scattered in the southeast, but these facilities were mainly concentrated on either side of the northwest ring road and near the main transportation space. Their distribution pattern was circular, following the circular road network (Figure 2).

#### 3.1.2 Analysis of spatial characteristics of COVID-19 outbreak frequency

In the analysis of the spatial characteristics of COVID-19 outbreak frequency, 500 m × 500 m rectangular cells covering the study area was established using ArcGIS, and the outbreak frequency within the rectangular cells was calculated based on the Beijing Municipal Government Data Resource Network, the Beijing Municipal Health and Wellness Commission, and Beijing Daily Public by recording information on the locations of the residential neighborhoods with new daily confirmed cases within Beijing’s fifth ring road from November 2021 to October 2022, as well as the locations of medium- and high-risk areas.

**Figure 3:**
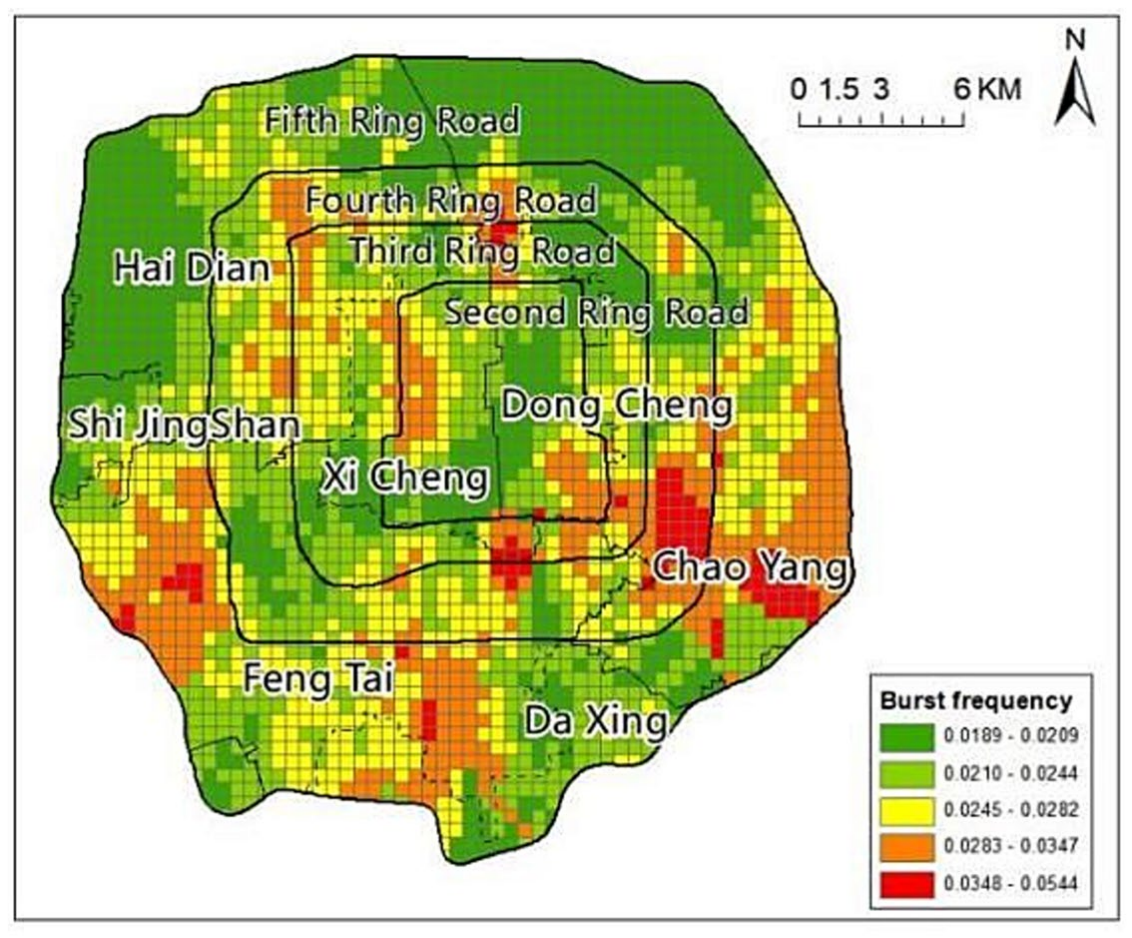
Spatial distribution of COVID-19 outbreak frequency.

In terms of its spatial pattern, outbreak frequency was lower in the central and peripheral segments and higher in the remaining segments of the study area. The highest outbreak frequencies were mainly concentrated in the areas outside the South Second Ring Road, with the highest in Chaoyang District, followed by Shijingshan and Daxing Districts, and scattered in the northwest part of Xicheng District and Haidian District (Figure 3).

#### 3.1.3 Geographically weighted regression analysis of COVID-19 outbreak frequency and external spatial environment

The global spatial autocorrelation analysis was conducted on the frequency of COVID-19 outbreaks in the study area, along with external spatial environmental indicators. Global spatial autocorrelation is a comprehensive measure of spatial data for the entire study region. It is used to reflect whether spatial data exhibits clustering or dispersal trends, as well as the strength and significance of these trends. The Moran Index is the ratio of covariance to variance, taking into account spatial location relationships, and it represents the spatial autocorrelation coefficient. Moran’s I index values can be bounded to the range − 1.0 to +1.0 when the weights are row standardized, An index score higher than 0.3 is an indication of relatively strong positive autocorrelation. A small value of p (usually *p* < 0.05) indicates that we can reject the null hypothesis of complete spatial randomness and accept that spatial autocorrelation exists, and z-score values are indicative and can be differentiated based on data (38). The results showed that the Moran’s I indexes of all indicators were more than 0.3, the z-score values were greater than 0, with a value of p less than 0.001, indicating that the global spatial autocorrelation of the outbreak frequency was highly significant and exhibits a strong positive correlation in space.

A test was conducted on the multicollinearity of six indicators in the study area: building density, plot ratio, water and green coverage, commercial facility density, public service facility density, and transportation facility density. Variance inflation factor (VIF) is a measure that assesses the severity of multicollinearity in a multiple linear regression model. It represents the ratio of the variance of the regression coefficient estimate to the variance assuming non-linear dependence between the independent variables. The VIF values for all indicators were less than 5, indicating that there is little or no multicollinearity problem (39) (Table 2).

**Table 2:** Global spatial autocorrelation significance and multiple collinearity test.

Projects	Moran’s I	*z*	*p*	VIF
The COVID-19 outbreak frequency	0.827	85.905	0.000	–
BD	0.465	48.328	0.000	1.508
VR	0.594	61.682	0.000	1.471
WGC	0.572	59.423	0.000	1.430
DCF	0.498	51.821	0.000	1.783
DSF	0.700	72.717	0.000	1.971
DTF	0.400	41.562	0.000	1.425

The COVID-19 outbreak frequency in a total of 2,769 rectangular cells of 500 m × 500 m within the study area was used as the dependent variable. Six indicators, including BD, VR, WGC, DCF, DSF, and DTF, were used as independent variables to construct a GWR model. The measured coefficients, R^2^ and adjusted R^2^, of the GWR model were 0.79 and 0.74, respectively. Both were greater than 0.7 and the absolute values of the regression coefficients of each element were large, indicating that the GWR model had a strong explanatory effect. There was a significant correlation between the frequency of epidemic outbreaks and the external spatial environment (Table 3).

**Table 3:** GWR model determination coefficients.

*R* ^2^	*R*^2^ adjusted	AICc	Residual squares	Bandwidth (km)
*0.792374*	*0.735484*	*−4862.462265*	*19.40998*	*1717.829009*
Projects	Average value	Median	Maximum value	Minimum value
Local *R*^2^	0.178944	0.168504	0.574433	0.008505
BD	0.093273	−0.061378	1.984351	−1.200134
VR	0.023199	0.024738	0.444783	−0.4181
WGC	−0.022804	−0.023266	0.584145	−0.543087
DCF	0.292249	0.053153	1.420713	−0.98085
DSF	0.036014	0.029701	3.324738	−3.515115
DTF	0.385493	0.082922	16.427417	−11.016806

The results of the local R^2^ and regression coefficients in the geographically weighted regression model show that the local R^2^ can explain the degree of fit of the model in different spatial areas, ranging from 0.009 to 0.574, with a large difference. This indicates significant spatial heterogeneity in the relationship between COVID-19 outbreak frequency and external spatial environmental factors. In terms of spatial distribution, high-value points are mostly clustered in the southwest of the central Xicheng District, the southern part of the Dongcheng District, as well as the western part of the Fengtai District, the southern part of the Daxing District, and the northwest part of the Chaoyang District.

**Figure 4:**
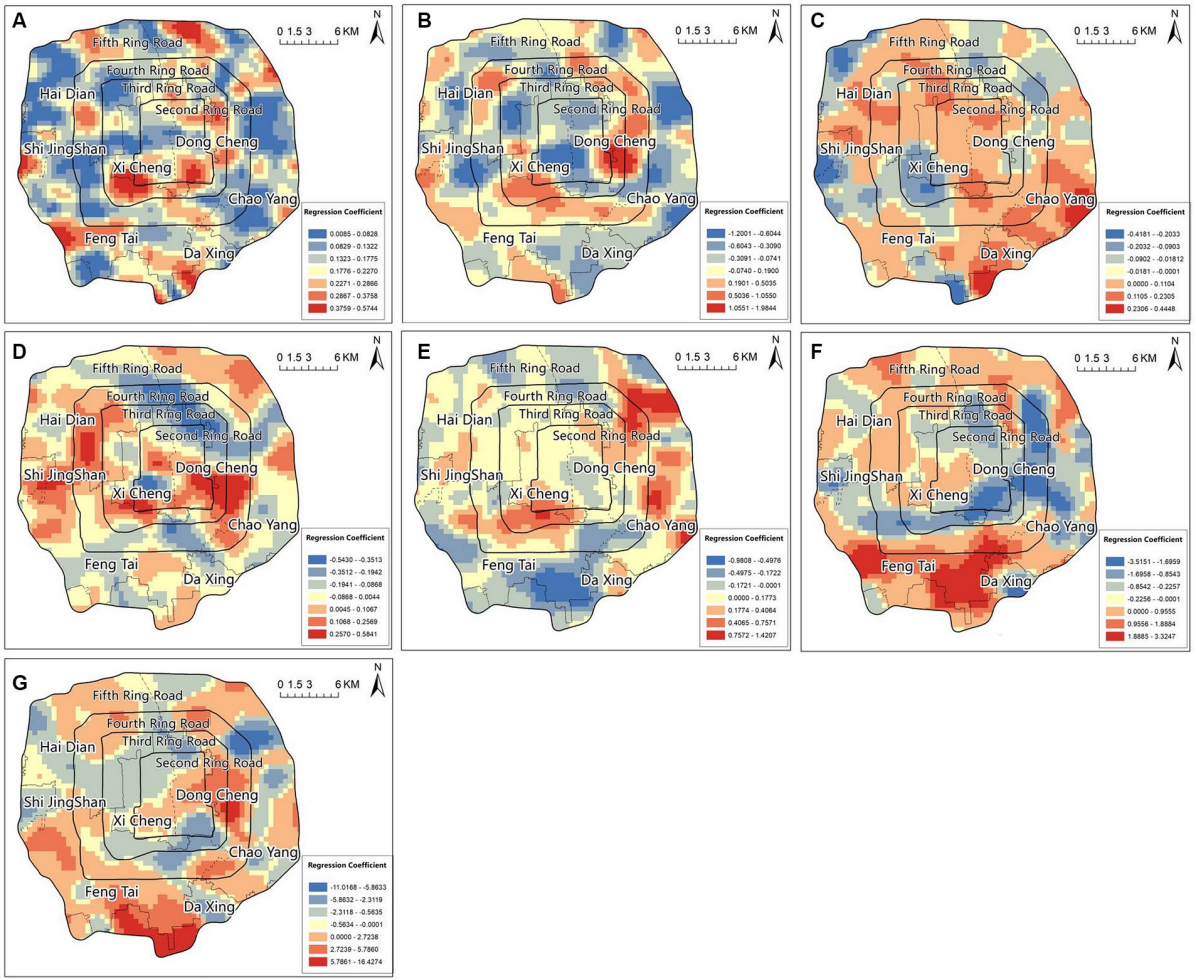
Geographically weighted regression model local R^2^ **(A)**, regression coefficients of BD **(B)**, VR **(C)**, WGC **(D)**, DCF **(E)**, DSF **(F)**, DTF **(G)**.

The regression coefficients represent the degree of influence of the indicators on COVID-19 outbreak frequency. Among them, the regression coefficients of BD, VR, DCF, DSF, and DTF were mostly positive in spatial distribution, with positive average values. This indicated a positive overall correlation between these five factors and COVID-19 outbreak frequency within the study area. The regression coefficient of BD had a significantly high-value area in the central part of the Dongcheng District, where both BD and outbreak frequency were high, indicating a significant positive correlation. The regression coefficients of VR were at a medium to high level in the central, northwest, and southeast parts of the study area, with extremely high values in the southern part of Daxing and the eastern part of Chaoyang, where both VR and COVID-19 outbreak frequency were high. The areas with extremely high values for the regression coefficient of DCF were mostly clustered in the northern and southern parts of the Chaoyang District and the southern part of the Xicheng District. The regression coefficients of DSF and DTF showed similar trends in spatial distribution, with extremely high values in the southern part of the study area. The regression coefficients of WGC were mainly negative, with negative average and median values, indicating a negative correlation between this factor and COVID-19 outbreak frequency within the study area. Low-value areas were mostly located in the area between the second and fifth ring roads in the northern part, and there was also an extremely low point in the central Xicheng District, where there were large parks (Figure 4).

**Figure 5:**
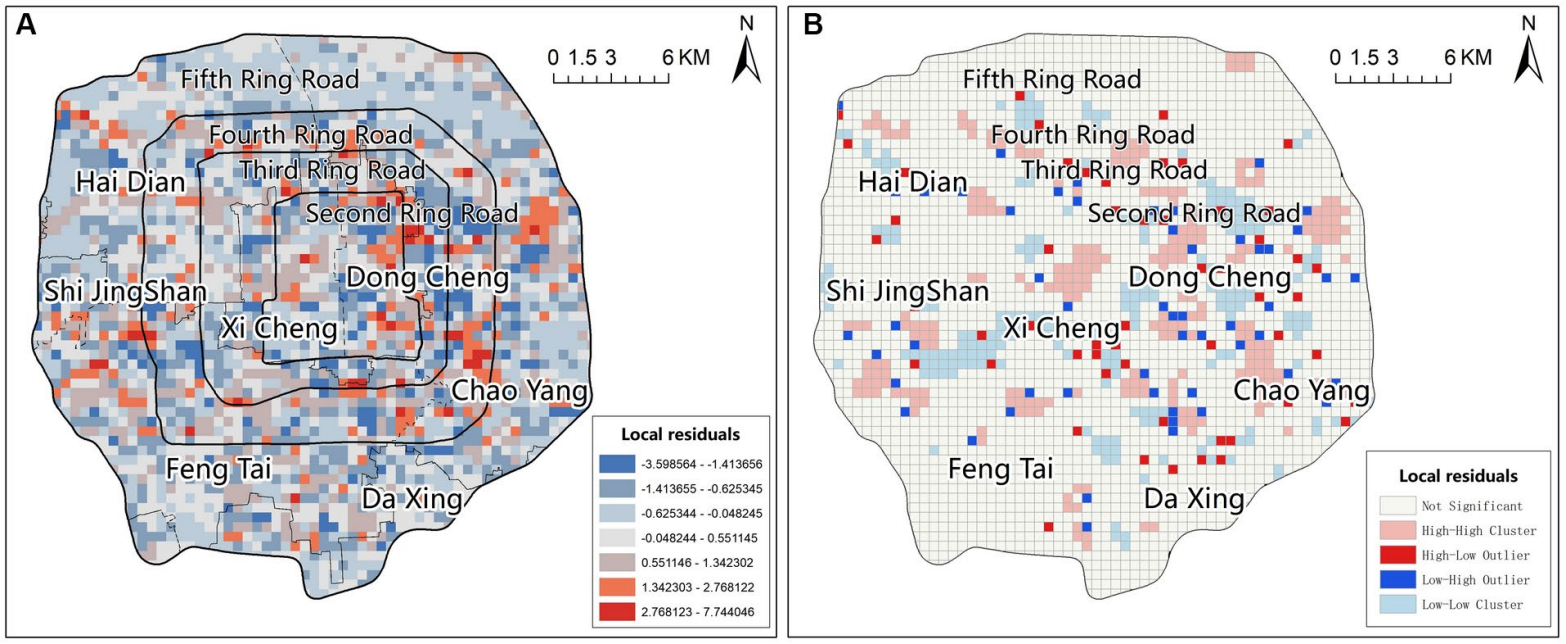
The spatial distribution of the standardized residues **(A)**, and the spatial autocorrelation **(B)**.

To better understand the regression effect of GWR, we plotted the spatial distribution map of standardized residuals and estimated spatial autocorrelation using local Moran’s I to track potential clustering in the residuals. The results indicated that the residuals were not significant in the majority of the areas and were randomly dispersed. This suggested that GWR had addressed the spatial heterogeneity issue in most locations. However, there were also some regions where clustering and spatial outliers were present, indicating that cells with significantly high residuals were adjacent to cells with significantly low residuals (Figure 5). This pattern had also been observed in other studies analyzing COVID-19 using spatial regression methods, which could be explained by the presence of spatial heterogeneity in certain areas or the need to include additional variables in the model (40, 41).

### 3.2 Correlation analysis of COVID-19 outbreak frequency and internal spatial environment

#### 3.2.1 Cluster analysis

Cluster analysis was mainly to divide the research objects into several clusters, and to conduct binary logistic regression for each cluster separately, in order to determine the correlation between different characteristics such as house prices, construction ages, number of buildings, number of households and the frequency of COVID-19 outbreak in communities. Using SPSS software, K-means cluster analysis was conducted for 344 epidemic-affected neighborhoods in the study area. K-means clustering is a commonly used partitioning clustering method that is efficient and easy to implement. Given a dataset and the desired number of clusters K, the user needs to specify the value of K, which represents the number of clusters. This algorithm, using various distance functions, iteratively calculates and assigns data points to K clusters automatically. We used four elements, including HP, BA, NB, and NH, to cluster the disease-related neighborhoods.

In order to determine the optimal number of clusters for our analysis, we employed the elbow method. We calculated the sum of squared errors (SSE) for a range of potential cluster numbers and plotted the SSE values against the number of clusters. By observing the plot, we identified the “elbow point” where the SSE started to level off. In our case, this occurred at a cluster number of 3, indicating that further increasing the number of clusters did not significantly reduce the SSE. Therefore, we concluded that 3 clusters were appropriate for our analysis. It is worth noting that the elbow method is a widely used approach for determining the optimal number of clusters, providing a balance between model complexity and goodness of fit.

The clustering results were obtained by K-means calculation. The final clustering centers for HP, BA, NB, and NH in the first cluster were at 54,892, 1,991, 18, and 1,904, respectively; in the second cluster, the clustering centers of HP, BA, NB, and NH were 81,800, 1,986, 14, and 1,392, respectively; in the third cluster, the clustering centers of HP, BA, NB, and NH were 107,399, 1,986, 16, and 1,204, respectively. In the three clusters, the mean differences were significantly different and showed an increasing trend, indicating that clusters 1, 2, and 3 characterized low-end, mid-end, and high-end neighborhoods, respectively. Based on the number of cases in each cluster, 185, 101, and 58 low-end, mid-end, and high-end neighborhoods, respectively, were involved in the epidemic within the study area.

The clustering results showed that low-end neighborhoods generally have lower HP, later BA, more NB and NH. Mid-end neighborhoods often have moderate HP and oNH, earlier BA, and fewer NB. High-end neighborhoods, on the other hand, generally have higher HP, earlier BA, more NB and fewer NH (Table 4).

**Table 4:** Final clustering centers.

	Clustering		
	1	2	3
HP	54,892	81,800	107,399
BA	1991	1986	1986
NB	18	14	16
NH	1904	1,392	1,204
**Distance**			
	1	2	3
1		26912.940	52511.492
2	26912.940		25599.441
3	52511.492	25599.441	
**Case items**			
1	185.000		
2	101.000		
3	58.000		
Effective	344.000		
Missing	0.000		

#### 3.2.2 Correlation analysis of COVID-19 outbreak frequency and internal spatial environment

Based on the results of the above cluster analysis, epidemic-affected neighborhoods in Beijing were classified into three classes: low-end, mid-end, and high-end. Their outbreak frequencies were analyzed separately to investigate the correlation between outbreak frequency in different classes of residential neighborhoods and four representative internal environmental factors, including HP, BA, NB, and NH.

(1) Correlation between COVID-19 outbreak frequency and the internal spatial environment in low-end neighborhoods

First, the COVID-19 outbreak frequency was assigned to the low-end neighborhoods according to the dichotomous method, and the neighborhoods where the COVID-19 outbreak frequency was greater than 0.019 was assigned a value of 1, and the opposite was assigned a value of 0. Second, the assigned Outbreak Frequency was logistically regressed with the above four representative internal environmental factors, and the results showed a significance of 0.730. This was greater than the comparative value of 0.05, indicating that there was no significant difference between the predicted and true values and the model fit was good (42).

Next, the regression equation model was established using the best predictive elements. A total of four urban spatial environmental factors affecting the probability of events were selected. After the model had been tested and screened, the significance of HP and NH in the model was <0.05, which passed the significance test and was entered into the equation (Table 5). We then concluded that the frequency of epidemic outbreaks in low-end neighborhoods was positively correlated with HP and the NH: the higher the HP and the larger the NH, the more frequent the epidemic outbreaks.

**Table 5:** Hosmer-Lemeshaw test and introduction of covariates in the model of low-end neighborhoods.

Card side		Degree of freedom		Significance		
5.258		8		0.730		
		B	Significance			Significance
Enter the equation elements	HP	0.000***	0.034	Not entered into the equation elements	BA	0.777
	NB	0.000***	0.037		NH	0.916
					Constants	0.936

Note: *** represents significance level of 1% respectively.

(2) Correlation between COVID-19 outbreak frequency and the internal spatial environment in mid-end neighborhoods

First, the COVID-19 outbreak frequency was assigned to the middle-grade neighborhoods according to the dichotomous method, and the neighborhoods where the COVID-19 outbreak frequency was greater than 0.019 was assigned a value of 1, and the opposite was assigned a value of 0. Second, the assigned Outbreak Frequency was logistically regressed against the above four representative internal environmental factors, and the results showed a significance of 0.853. This is greater than the comparative value of 0.05, indicating that there was no significant difference between the predicted and true values and the model fit was good.

Next, the regression equation model was established using the best predictive elements. A total of four urban spatial environmental elements that affected the probability of events were selected. After the model had been tested and screened, the significance of the NH in the model was <0.05, which passed the significance test and was entered into the equation (Table 6). We concluded that the frequency of epidemic outbreaks in mid-range neighborhoods was positively correlated with the NH: the more households, the more COVID-19 outbreak frequency.

**Table 6:** Hosmer-Lemeshaw test and Introduction of covariates in the model of mid-end neighborhoods.

Card side		Degree of freedom		Significance		
4.043		8		0.853		
		B	Significance			Significance
Enter the equation elements	NB	0.001***	0.035	Not entered into the equation elements	HP	0.718
					BA	0.552
					NH	0.543
					Constants	0.555

Note: *** represents significance level of 1% respectively.

(3) Correlation between COVID-19 outbreak frequency and the internal spatial environment in high-end neighborhoods

First, epidemic COVID-19 outbreak frequency was assigned to cluster 1 if it was greater than 0.019 and 0 if it was greater than 0.019. Second, the assigned COVID-19 outbreak frequency was logistically regressed against the above four representative internal environmental factors, and the results showed a significance of 0.036 (Table 7). As this is less than the comparative value of 0.05, we concluded that there was a significant difference between the predicted and true values and the model fit was poor. Therefore, there was no significant correlation between the COVID-19 outbreak frequency and the HP, BA, NB, and NH in high-end neighborhoods.

**Table 7:** Hosmer-Lemeshaw test of high-end neighborhoods.

Card side	Degree of freedom	Significance
16.518	8	0.036

## 4 Discussion

### 4.1 Correlation of COVID-19 outbreak frequency and external spatial environment from the perspective of spatial justice

In regards to the correlation analysis between the COVID-19 outbreak frequency and urban external spatial environment, GWR regression results indicated a significant correlation between the urban spatial environmental factors and COVID-19 outbreak frequency. Building density, volume ratio, density of commercial facilities, density of service facilities, and density of transportation facilities showed a positive correlation with COVID-19 outbreak frequency, as these factors can increase population mobility and contact opportunities, therefore increasing the risk of disease spread. Water and green coverage had a negative correlation with epidemic outbreaks, as they provided better air quality, reduced pollution, and the opportunity for disease spread.

From the perspective of spatial justice, these research results remind us of the need to consider principles of fairness and sustainable development in urban planning and development. Urban spatial environmental planning should strive to achieve a fair distribution of resources, ensuring that all residents can enjoy good living conditions and a healthy environment. For example, water and green coverage have a significant negative impact on epidemic outbreaks, so increasing water and green coverage in high-density building areas can improve air and environmental quality.

Studies have shown that urban green space planning is particularly important for inhibiting the spread and diffusion of epidemics in cities, and establishing an integrated green open space system can effectively improve urban environments, enhancing the ability of cities to respond to epidemics and climate natural disasters (43, 44). On the other hand, even with restrictions caused by epidemics, people’s demand for outdoor green spaces is still significant, and landscapes and plants can relieve users’ moods and have a positive effect on post-illness recovery (45–47).

Compared with previous studies, this research focuses on the green and water coverage extracted from satellite imagery. While previous studies primarily relied on park greenery points of interest (POI) (48, 49), the findings of this study demonstrate that in addition to park green spaces, environmental factors such as road greening and community greening also have a positive effect on impeding the spread of epidemics. Therefore, attention should be given to spatial justice issues regarding green infrastructure, as well as the planning, design, and utilization of informal blue-green spaces outside of parks. By narrowing the inequalities in urban greening, it is possible to ensure equal opportunities for urban residents to enjoy green resources and establish a balanced, inclusive, hierarchically classified, highly accessible, and efficient blue-green space system (50).

Places where people gather in large numbers, such as commercial and service facilities, are more likely to promote the spread of COVID-19, often becoming the first outbreak site, and leading to pandemics in other areas of the city due to population density (51, 52). Regions with high building density and floor area ratio also tend to create population density, and the higher the aggregation degree of urban space, the more likely it is to be an enclosed space with poor ventilation, which promotes the spread of viruses (53, 54). The city’s vulnerability to various external shocks, especially sudden shocks, increases and urban safety risks become higher.

The spatial distribution of facility distribution and construction density in Beijing addressed in this study was highly uneven, resulting in a non-uniform spatial distribution of population. This not only affected the daily living experience of residents, but also had a significant adverse impact on epidemic prevention and control (55, 56).

In the central urban area within the second ring road of Beijing, the living style adopted a high-density hutong-like residential form. In this living style, the community had narrow roads and dense housing, and there might be facilities with a high risk of transmission, such as shared kitchens and public toilets. This undoubtedly posed challenges to epidemic prevention and control. On the other hand, being a vast city, Beijing attracted a large number of migrants, and the number of migrants had been increasing year after year. Influenced by high housing prices, many migrants chose to rent housing, often in the form of shared apartments with multiple occupants. This led to the concentration of populations in relatively small living spaces, facilitating the spread of epidemics (57).

Furthermore, some studies have found that in the short term, epidemic occurrences and spread are often associated with large-scale commercial markets. These markets typically involved significant movements of people and goods (58–60). Particularly as the core city in the Beijing-Tianjin-Hebei urban agglomeration, large-scale commercial markets and transportation hubs in Beijing became centers for the dispersal of people and goods in the metropolitan area, resulting in a high frequency and rapid spread of epidemics over a wide area (61, 62). Therefore, it is important to pay attention to the popularization of public service facilities, fully consider the needs and accessibility of residents in the layout of facilities, reduce high-density urban development, and avoid the concentration of human flow. This can reduce the inequality of resources and services and improve the public welfare of residents.

### 4.2 Correlation of COVID-19 outbreak frequency and internal spatial environment from the perspective of spatial justice

According to the analysis of the correlation between the COVID-19 outbreak frequency and the internal spatial environment of cities, the COVID-19 outbreak frequency is significantly correlated with the internal environment of low-end and mid-end, while it is not significantly correlated with the internal environment of high-end. This may mean that there are some factors in the internal environment of low-end and mid-end that are not conducive to interrupting the transmission of diseases.

The key to understanding spatial justice is recognizing that the unequal distribution of spatial resources and services within cities can lead to inequality among social groups. In this case, low-end and mid-end may face a higher risk of epidemic outbreaks because they may not have access to the favorable internal environmental conditions found in high-end (63, 64). Research has shown that the age of community construction can affect the degree of spatial openness, with older communities being much more exposed than newly built ones. Characteristics such as the age, quality, property level, and spatial environment of a community can be used to differentiate between different grades, and thus their correlation with the frequency of epidemic outbreaks may vary (65–67).

Many existing studies indicate that economic inequality between communities often leads to disparities in material spatial environment and access to public infrastructure and services, resulting in neighborhood deprivation. This phenomenon is manifested in communities of different races, ages, genders, and economic structures (68–72). Compared to other related studies focusing on the urban environmental impact of epidemics in developing countries and regions in Asia, Africa, and Latin America, this study chose Beijing, which is a relatively developed city, but still suffers from unbalanced urban space and community development. As a developing country’s city, research on Beijing could illustrate that income gaps among residents have caused spatial residential differentiation, resulting in differences in the spatial environment within and surrounding different income groups’ residential areas. This might even affect the resilience and robustness of different income groups to epidemics.

The COVID-19 outbreak frequency in low-end is positively correlated with housing prices and the number of households. This may be due to the fact that low-end are often located in remote areas, where the population consists mainly of low-income individuals who need to commute for longer distances. As the distance and commuting time increase while the housing prices decrease, the number of households and population density relative to the area may decrease, thus reducing the likelihood of residents getting infected and leading to lower outbreak frequencies. The COVID-19 outbreak frequency in medium-end is positively correlated with the number of households and population density. The more households there are within a neighborhood, the higher the population density becomes. Since the primary transmission pathways of COVID-19 are related to gatherings in households and various types of public spaces, an increase in the number of households leads to higher population mobility and frequency of gathering in public places, resulting in higher outbreak frequencies. On the other hand, the COVID-19 outbreak frequency in high-end showed no significant correlation with the internal environment, possibly due to stricter control measures and relatively well-equipped healthcare facilities, which effectively prevent and control the outbreak of epidemics.

The conclusions of this study can provide new insights for epidemic prevention and control in densely populated developing countries, including Beijing, China, and even in Asia. In order to achieve spatial justice, society should strive to address the inequality phenomena of income gaps and residential differentiation, and promote fair distribution of spatial environments. This includes providing sufficient medical resources and healthcare facilities, improving internal environmental conditions in low-income and middle-income communities, reducing the risk of disease outbreaks, and ensuring that all social groups have equal access to urban spatial resources and services.

## 5 Conclusion

This study used Geographically Weighted Regression, cluster analysis, Binomial Logistic Regression, and BP neural network to investigate the correlation between COVID-19 outbreak frequency and the internal and external spatial environment of cities. We drew the following conclusions.

Firstly, the results of the GWR showed that the COVID-19 outbreak frequency was evidently correlated with the external spatial environment of the city. Elements of the urban external spatial environment, such as building density, volume ratio, density of commercial facilities, density of service facilities, and density of transportation facilities, were positively correlated with COVID-19 outbreak frequency, while water and greenery coverage was negatively correlated with COVID-19 outbreak frequency.

Secondly, the correlation between the COVID-19 outbreak frequency and the internal spatial environmental elements of the neighborhood varied among different grades of neighborhoods. The COVID-19 outbreak frequency in low-end neighborhoods was significantly correlated with the internal spatial environmental factors, among which house price and the number of households were positively correlated with the COVID-19 outbreak frequency. The higher the house prices and the more households in low-end neighborhoods, the more frequent the epidemic outbreaks. The COVID-19 outbreak frequency in mid-end neighborhoods was significantly correlated with internal spatial environmental factors. The number of households in mid-end neighborhoods was positively correlated with the COVID-19 outbreak frequency. The larger the number of households, the more frequent the epidemic outbreaks. There was no significant correlation between the COVID-19 outbreak frequency in high-end neighborhoods and the internal spatial environmental elements of these neighborhoods.

Finally, to optimize urban spatial environment and reduce the spread of the COVID-19 pandemic, measures can be taken in terms of density, green spaces, public service facilities.

Density: Implement urban planning policies to promote balanced population density distribution and avoid overcrowding in certain areas. Encourage mixed land-use development to create diverse and well-connected. Improve transportation infrastructure to reduce congestion and crowded public spaces. Promote remote work and flexible working hours to reduce commuting and crowded public transportation.

Green Spaces: Increase the availability and accessibility of green spaces, such as parks, gardens, and urban forests, in all. Enhance the maintenance and cleanliness of existing green spaces to encourage their utilization by residents. Promote rooftop gardens and vertical greenery to optimize limited urban space.

Public Service Facilities: Ensure equitable distribution of essential public service facilities, such as healthcare centers, hospitals, and testing centers, across all. Improve the capacity and efficiency of healthcare facilities to handle a surge in the number of cases. Enhance sanitation and hygiene infrastructure, including public restrooms and handwashing stations, especially in densely populated areas.

These measures can contribute to optimizing the urban spatial environment, reducing the transmission of the COVID-19 pandemic, and promoting more equitable and resilient urban communities.

## Data availability statement

The raw data supporting the conclusions of this article will be made available by the authors, without undue reservation.

## Author contributions

ZY: Conceptualization, Funding acquisition, Methodology, Writing – review & editing. JXL: Conceptualization, Formal analysis, Methodology, Software, Writing – original draft. YLi: Conceptualization, Funding acquisition, Methodology, Writing – review & editing. XWH: Data curation, Formal analysis, Investigation, Software, Writing – original draft. AZ: Data curation, Formal analysis, Investigation, Software, Writing – original draft. YLu: Data curation, Formal analysis, Investigation, Software, Writing – original draft. XZ: Formal analysis, Supervision, Writing – original draft. XYY: Formal analysis, Writing – original draft.

## Footnotes

^1^ https://data.beijing.gov.cn/index.htm

^2^ http://wjw.beijing.gov.cn/wjwh/ztzl/xxgzbd/gzbdyqtb/index.html

^3^ https://www.bjd.com.cn/index.shtml

^4^ https://www.amap.com/

^5^ https://www.gscloud.cn/
